# Factors associated with smoking cessation among adolescents: A retrospective study in a smoking cessation outpatient clinic

**DOI:** 10.18332/tid/221181

**Published:** 2026-07-10

**Authors:** Saliha Büşra Aksu, Güzin Zeren Öztürk, Beray Gelmez Taş

**Affiliations:** 1 Health Sciences University, Şişli Hamidiye Etfal Training and Research Hospital, İstanbul, Türkiye

**Keywords:** smoking cessation, adolescent, tobacco products, smoking cessation agents, adolescent health

## Abstract

**INTRODUCTION:**

This study aimed to evaluate smoking cessation status and factors associated with cessation among individuals aged ≤20 years who attended a smoking cessation outpatient clinic during the first month of follow-up.

**METHODS:**

This was a single-center, descriptive study conducted among adolescents who applied to a Smoking Cessation Outpatient Clinic. Patients’ sociodemographic characteristics and smoking histories were recorded, and nicotine dependence was assessed using the Fagerström test for nicotine dependence (FTND). Statistical analyses included descriptive statistics, group comparisons using appropriate parametric or nonparametric tests, and multivariable logistic regression.

**RESULTS:**

A total of 229 adolescents were included. At one month, the overall cessation rate was 7.9% (n=18). At one month, adolescents who quit smoking had lower daily cigarette consumption and lower nicotine dependence scores. In multivariable analysis, only the FTND score remained independently associated with cessation (AOR=0.807; 95% CI: 0.660–0.987).

**CONCLUSIONS:**

Among Turkish adolescents seeking smoking cessation support, lower nicotine dependence was the primary predictor of short-term quitting success. For public health policies, early identification and timely intervention in adolescent smoking are essential to reduce the potential long-term health risks and future healthcare costs, particularly given the increasingly early age of smoking initiation.

## INTRODUCTION

Smoking remains a major global public health problem. Tobacco use is responsible for at least 12 different types of cancer. It accounts for 80–90% of lung cancer-related deaths, making it one of the leading risk factors for preventable disease and premature mortality^1^. According to the World Health Organization (WHO), more than 8 million people die each year due to tobacco use^2^. Data from the Turkish Statistical Institute (TUIK) indicate that 28.3% of individuals aged ≥15 years use tobacco products daily, and among those aged 15–24 years, daily tobacco use increased from 14.3% in 2012 to 19.3% in 2022^3^. In contrast, countries such as the United Kingdom and the United States have reported substantial declines in adolescent cigarette use over the past decade, largely attributed to comprehensive tobacco control policies and strong school-based prevention programs^4,5^. This rising trend among young people represents a critical public health concern with significant long-term consequences.

Adolescents are particularly vulnerable to addictive behaviors due to ongoing maturation of reward-processing and impulse-control circuits, which heightens sensitivity to nicotine and increases the likelihood that even early or low-dose exposure will lead to dependence^6-8^. At the same time, experimentation with novel or risky behaviors is a normative part of adolescent development, further facilitating early initiation^9^. Smoking during adolescence is also associated with mental health problems such as depression and anxiety, and is frequently linked to other high-risk behaviors^10^. Most adult smokers begin smoking before the age of 18 years, and early initiation is strongly associated with higher nicotine dependence and reduced cessation success later in life^11,12^. Furthermore, early smoking initiation increases the risk of chronic diseases such as cardiovascular disorders, respiratory illnesses, and cancer^2^.

Despite these risks, evidence-based smoking cessation treatments specifically targeting adolescents remain limited. Recent systematic reviews have underscored the scarcity of effective interventions for individuals under 20 years of age and highlighted the urgent need for innovative, youth-oriented cessation strategies^13^. Evidence suggests that multicomponent behavioral interventions – such as school-based programs, motivational interviewing, text-messaging support, and family-involved counseling – are the most effective cessation strategies for adolescents^14-16^. These gaps in effective youth-focused cessation services highlight the need for adolescent-specific components within national tobacco control strategies, including strengthened screening, early identification of nicotine dependence, and accessible age-appropriate cessation support.

Therefore, this study aimed to investigate factors associated with smoking cessation among adolescents who attended a smoking cessation outpatient clinic. The findings are expected to contribute to the development of age-specific tobacco control policies and strengthen cessation support for this vulnerable group.

## METHODS

### Study design and population

This retrospective, descriptive, single-center study was conducted at a Ministry of Health–affiliated smoking-cessation clinic in Istanbul, Türkiye. Patients can access the clinic either through self-referral or referral from primary care physicians or other outpatient clinics. Certified smoking cessation clinics provide free behavioral counseling and pharmacotherapy to eligible individuals of all ages, including adolescents (with parental consent), under nationally regulated service protocols.

**Figure 1:**
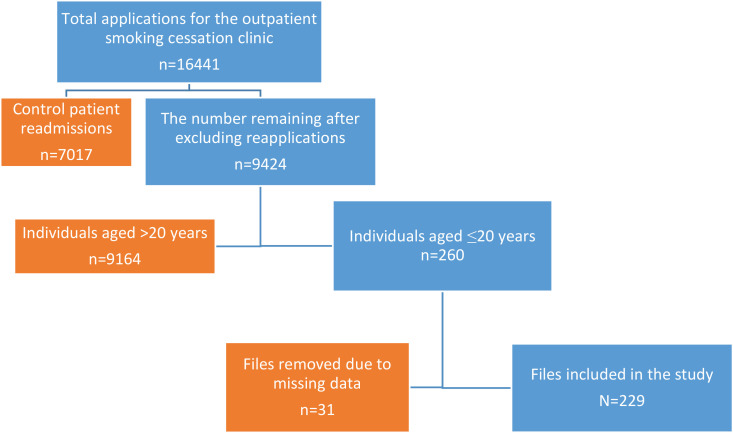
Flowchart illustrating the process of accessing patient files and selecting eligible adolescents for inclusion in the study, Türkiye, 2019–2022

During the study period (January 2019–December 2022), 16441 visits were recorded at the clinic. After excluding repeat visits and incomplete files, 9424 unique patient records remained. Of these, 260 patients were aged ≤20 years. After further excluding those with missing treatment data, incomplete Fagerström test for nicotine dependence (FTND) scores, or without follow-up visits, 229 patients were included in the final analysis (Figure 1). This ensured that only patients with complete baseline and follow-up data were included in the analysis. Ethical committee approval was obtained from Sisli Hamidiye Etfal Training and Research Hospital Clinical Research Ethics Committee protocol dated 13.06.2023 and numbered 3974.

At the first visit, all adolescents underwent a standardized clinical assessment that included sociodemographic characteristics, smoking history, daily cigarette consumption, nicotine dependence (FTND score), and motivation to quit. Behavioral counseling was provided to all patients by physicians certified in smoking-cessation treatment. Core components included education about nicotine withdrawal and craving patterns, identification of personal smoking triggers, and development of individualized coping strategies. Adolescents were encouraged to anticipate situations that increase relapse risk, such as stress, peer smoking, or boredom, and to use practical coping skills (e.g. behavioral distraction, oral substitutes, physical activity, or establishing smoke-free environments). Supportive counseling techniques were used throughout the process, including reinforcing the patient’s confidence in quitting, exploring motivation, addressing concerns, and offering ongoing encouragement.

Treatment decisions followed the national smoking cessation guideline of the Turkish Ministry of Health, supplemented by clinical judgment. Behavioral counseling constituted the standard intervention for all adolescents. At the same time, the decision to initiate pharmacotherapy or nicotine replacement therapy (NRT) was individualized based on age, clinical characteristics, and degree of nicotine dependence. According to national recommendations^17^, prescription medications (bupropion and varenicline) are not recommended for individuals aged <18 years and were therefore not used in this group. NRT (gum, patch, lozenge) was considered on a case-by-case basis for patients aged <18 years, depending on dependence severity and overall clinical suitability^17,18^. In contrast, adolescents aged ≥18 years were eligible for both NRT and prescription medications when clinically appropriate.

In routine practice, both nicotine replacement therapy (NRT) and prescription medications are generally prescribed for a minimum of two months. All counseling sessions were conducted individually. The study included only patients who attended the one-month follow-up and had received at least two face-to-face clinical consultations within that period, ensuring a minimum level of engagement with the treatment protocol. Smoking status at one month was determined retrospectively from patients’ self-reported abstinence, as recorded in the hospital information system.

### Data collection

Extracted variables included age, sex, education level, family income level, daily cigarette consumption, and FTND score. All data were obtained from electronic medical records and were fully anonymized before analysis, with no identifiable personal information collected or stored. Family income was categorized according to national standards; adolescents from households with a monthly income below the national minimum wage were classified as having low family income. The FTND was also collected, which consists of six items, with a total score ranging from 0 to 10. Higher scores indicate greater nicotine dependence^19^.

### Statistical analysis

Data analysis was conducted using IBM SPSS Statistics version 20.0. The normality of continuous variables was assessed with the Kolmogorov–Smirnov test. For comparisons between two independent groups, the Student’s t-test was used for normally distributed variables and the Mann–Whitney U test for non-normally distributed variables. Differences in categorical variables were analyzed using the chi-squared test. Multivariable logistic regression was performed to examine variables associated with smoking cessation at the follow-up at one month. Model fit was assessed using the Hosmer–Lemeshow goodness-of-fit test. Variables with a p<0.20 in the univariable analyses were included in the multivariable logistic regression model; the final multivariable model included the FTND score and pack-years. Interaction terms were not evaluated due to the limited number of cessation events and concerns regarding model overfitting. Because this study was retrospective and included all eligible adolescent patients within the study period, an a priori sample size calculation was not performed. All statistical tests were two-tailed. Statistical significance was set at p≤0.05.

## RESULTS

A total of 229 adolescents aged ≤20 years were included in the study, accounting for 2.75% of all patient records reviewed during the study period. The mean age ± standard deviation was 17.73 ± 1.23 years (range: 15–20), of whom 43 (18.8%) were younger than 18 years, and 161 (70.3%) were male. More than half of the participants (n=131; 57.2%) reported low family income. All participants were daily cigarette smokers, with a mean consumption of 13.46 ± 5.74 cigarettes per day. Overall, 146 participants (63.8%) smoked fewer than one pack per day (<20 cigarettes). The mean Fagerström test for nicotine dependence (FTND) score was 5.53 ± 2.56.

At the follow-up at one month, 18 participants (7.9%) reported quitting smoking. The association between participants’ smoking-cessation status at 1 month and their sociodemographic characteristics is shown in Table 1. Of the total sample, 144 individuals (62.9%) received pharmacotherapy in addition to behavioral counseling, whereas the remaining participants received behavioral counseling only. Comparisons of smoking habits and treatment characteristics between quitters and non-quitters are shown in Table 2.

**Table 1:** Sociodemographic characteristics of adolescents attending a smoking cessation outpatient clinic in Türkiye, January 2019–December 2022 (N=229)

*Variables*	*Quitters* *(N=18)* *n (%)*	*Non-quitters* *(N=211)* *n (%)*	*p*
**Age** (years), mean ± SD	19.05 ± 1.21	18.70 ± 1.34	0.287^a^
**Gender**			0.725^b^
Female	6 (8.8)	62 (91.2)	
Male	12 (7.5)	149 (92.5)	
**Income level**			0.254^b^
Low	8 (6.1)	123 (93.9)	
High	10 (10.2)	88 (89.8)	

a Mann–Whitney U test.

b Chi-squared test.

**Table 2:** Comparison of smoking-related characteristics and treatment modalities, by smoking cessation status at one month, among adolescents attending a smoking cessation outpatient clinic in Türkiye, January 2019–December 2022 (N=229)

*Variables*	*Quitters* *(N=18)* *n (%)*	*Non-quitters* *(N=211)* *n (%)*	*p*
FTND, mean ± SD	4.22 ± 2.23	5.68 ± 2.59	**0.012**
Pack-years, mean ± SD	4.68 ± 6.07	6.28 ± 7.37	**0.045**
**Treatment modalities**			0.254
Behavioral therapy only	6 (7.1)	79 (92.9)	
NRT+ behavioral therapy	9 (10.0)	81 (90.0)	
Pharmacotherapy + behavioral therapy	3 (5.6)	51 (94.4)	

FTND: Fagerström test for nicotine dependence. NRT: Nicotine replacement therapy.

**Table 3:** Multivariable logistic regression analysis of factors associated with smoking cessation at one month, among adolescents attending a smoking cessation outpatient clinic in Türkiye, January 2019–December 2022 (N=229)

*Variable*	*AOR*	*95% CI*	*p*
FTND score	0.807	0.660–0.987	**0.037**
Pack-years	0.990	0.906–1.082	0.824

AOR: adjusted odds ratio. FTND: Fagerström test for nicotine dependence. Variables with p<0.20 in univariable analyses were included in the multivariable model. Model fit was acceptable according to the Hosmer–Lemeshow test (χ²=8.02; df=8, p=0.432).

In the multivariable logistic regression model, only the FTND score remained independently associated with smoking cessation at one month. The Hosmer–Lemeshow goodness-of-fit test indicated acceptable model fit (χ^2^=8.02, df=8, p=0.432). In the multivariable model, higher FTND scores were associated with lower odds of smoking cessation (OR=0.807; 95% CI: 0.660–0.987; p=0.037). The association between pack-years and smoking cessation was not statistically significant in the multivariable model (p>0.05).

## DISCUSSION

This study evaluated smoking cessation rates and associated factors among adolescents aged ≤20 years who attended a smoking cessation outpatient clinic. The overall one-month cessation rate was 7.9%. In multivariable analysis, only the Fagerström test for nicotine dependence (FTND) score remained independently associated with smoking cessation, indicating that lower nicotine dependence increased the likelihood of short-term quitting. Consistent with previous studies^20^, our findings confirm that adolescents with lower FTND scores and lower daily cigarette consumption were more likely to achieve abstinence. In our sample, 51.1% had high nicotine dependence, representing a substantial barrier to quitting^21^.

National data indicate that 28% of adolescents aged 11–19 years are current smokers^22^, with initiation often occurring between ages 6 and 12 years^23^. Nearly 80% of youth who begin smoking continue into adulthood, and one-third are expected to die prematurely from smoking-related diseases^13^. Early intervention is therefore essential, particularly for adolescents with low dependence who may benefit most from targeted cessation support. In our study, however, adolescents aged >20 years accounted for only 2.75% of all smoking cessation clinic visits. This underrepresentation may reflect barriers such as concealment of smoking habits from parents, limited adolescent-oriented cessation services, and the parental consent requirement for those aged <18 years.

Our one-month cessation rate of 7.9% was lower than the 12.2% reported in a US national survey of daily-smoking adolescents attempting to quit^24^. Similar short-term quit rates of 7–12% have been observed in school-based programs in North America and Europe, with higher success in structured, adolescent-focused interventions^25^. Service delivery differences may partly explain these disparities. In the US, school-based programs, youth quitlines, and text-messaging support are common, whereas in Türkiye, services are predominantly clinic-based and designed for adults.

In fact, the outcomes in our study more closely resemble those seen in unassisted or minimally assisted quit attempts, where quit rates among adolescents often fall below 10%^24^. A European multi-country survey reported that 50.1% of adolescent smokers had attempted to quit, with about half achieving short-term abstinence^26^. This contrast underscores the unique challenges faced by adolescents seeking cessation support in Türkiye. Compared with participants in structured interventions, adolescents attending routine clinics may present with higher nicotine dependence, more complex psychosocial barriers, or lower readiness to quit. Additionally, the absence of youth-specific cessation services and the restrictions on pharmacotherapy use in individuals aged <18 years likely contribute to the comparatively lower short-term cessation outcomes observed in our cohort.

These challenges are further compounded by structural limitations in the national cessation framework. Türkiye currently lacks a national clinical guideline specifically addressing smoking cessation in adolescents, and existing Ministry of Health guidelines do not provide recommendations for NRT use in individuals under 18 years of age^17^. In addition, NRT products available on the Turkish market do not include guidance for use in this age group in their package inserts. Together, these clinical and regulatory gaps may constrain clinicians’ treatment options and contribute to the relatively low cessation rates observed among adolescents in routine clinical settings.

In terms of treatment approaches, pharmacotherapy combined with behavioral counseling did not significantly improve overall quit rates in our study. However, success rates were higher among those aged ≥18 years (68.8%) compared with younger adolescents (50%), suggesting that pharmacological interventions may be more effective in older adolescents. While the short-term safety of nicotine replacement therapy (NRT) in adolescents has been demonstrated^27^, its effectiveness in long-term cessation remains limited^28^. This underlines the importance of cautious use of pharmacotherapy and prioritization of behavioral interventions in younger patients.

Tobacco product use among adolescents is also evolving, with increasing prevalence of electronic cigarettes and hookah^29^. Although often marketed as harm-reduction tools, adolescent e-cigarette use is linked to an increased likelihood of cigarette smoking initiation within 30 days^30^, and its long-term health consequences remain uncertain^31^. Hookah use, in particular, has been associated with cardiovascular risks^32^. These findings emphasize the importance of comprehensive screening for all tobacco products – not only cigarettes – during adolescent health visits. Counseling should also address secondhand smoke exposure, a well-established cause of respiratory and systemic disease^33^. Family and peer influences are particularly important; parental smoking strongly impacts adolescent behavior^34^, while peer smoking remains a critical driver of initiation^34^.

From a public health perspective, primary care and school health services provide valuable opportunities for early identification and intervention. However, smoking status is not consistently assessed in adolescent consultations, and disclosure is less likely in the presence of parents^35^. Confidential screening and counseling, along with routine integration of tobacco use questions into adolescent health visits, are recommended. School-based health education should address peer and family influences, while community-level strategies can reinforce non-smoking norms. In addition, digital and mobile health interventions, such as text message-based cessation support, have shown promising quit rates of up to 31% in adolescents^36^ and could be adapted for the Turkish context.

### Limitations

This study has several limitations. The small sample size and limited number of cessation events reduced statistical power and constrained the complexity of the multivariable model. Because of the retrospective design, the findings represent associations rather than causal relationships, and residual confounding may remain despite adjustment. The use of data from a single outpatient clinic may limit generalizability to adolescents in other settings or countries. In addition, cessation outcomes relied on self-report without biochemical verification, which may introduce misclassification bias. Also, pack-years, a measure originally developed for adult smoking exposure, may not optimally capture smoking patterns in adolescents with shorter smoking histories and should be interpreted cautiously.

## CONCLUSIONS

Among Turkish adolescents seeking smoking cessation support, lower nicotine dependence was the primary predictor of short-term quitting success. Compared with international adolescent populations, quit rates in this group remained modest, reflecting gaps in accessibility and intervention models. These findings suggest that, beyond individual-level factors such as nicotine dependence, broader contextual and structural gaps in accessibility and youth-oriented intervention models may contribute to poorer cessation outcomes.

Implementing culturally tailored, confidential, and school-linked cessation programs – especially for early-stage smokers – could improve outcomes and reduce the long-term health burden of tobacco use in this vulnerable population. Preventive strategies should prioritize identifying adolescents at an early stage of smoking, before high nicotine dependence develops, and integrating school-based, digital, and community approaches into comprehensive tobacco control policies.

## Data Availability

The data supporting this research are available from the authors on reasonable request.
